# Performances of Whole Tumor Texture Analysis Based on MRI: Predicting Preoperative T Stage of Rectal Carcinomas

**DOI:** 10.3389/fonc.2021.678441

**Published:** 2021-08-03

**Authors:** Jia You, Jiandong Yin

**Affiliations:** Department of Radiology, Shengjing Hospital of China Medical University, Shenyang, China

**Keywords:** magnetic resonance imaging, rectal cancer, apparent diffusion coefficient, texture analysis, T stage

## Abstract

**Objective:**

To determine whether there is a correlation between texture features extracted from high-resolution T2-weighted imaging (HR-T2WI) or apparent diffusion coefficient (ADC) maps and the preoperative T stage (stages T1–2 *versus* T3–4) in rectal carcinomas.

**Materials and Methods:**

One hundred and fifty four patients with rectal carcinomas who underwent preoperative HR-T2WI and diffusion-weighted imaging were enrolled. Patients were divided into training (n = 89) and validation (n = 65) cohorts. 3D Slicer was used to segment the entire volume of interest for whole tumors based on HR-T2WI and ADC maps. The least absolute shrinkage and selection operator (LASSO) was performed to select feature. The significantly difference was tested by the independent sample t-test and Mann-Whitney U test. The support vector machine (SVM) model was used to develop classification models. The correlation between features and T stage was assessed by Spearman’s correlation analysis. Multivariate logistic regression analysis was performed to identify independent predictors of tumor invasion. The performance of classifiers was evaluated by the receiver operating characteristic (ROC) curves.

**Results:**

The wavelet HHH NGTDM strength (*R_S_* = -0.364, *P* < 0.001) from HR-T2WI was an independent predictor of stage T3–4 tumors. The shape maximum 2D diameter column (*R_S_* = 0.431, *P* < 0.001), log σ = 5.0 mm 3D first-order maximum (*R_S_* = 0.276, *P* = 0.009), and log σ = 5.0 mm 3D first-order interquartile range (*R_S_* = -0.229, *P* = 0.032) from ADC maps were independent predictors. In training cohorts, the classification models from HR-T2WI, ADC maps and the combination of two achieved the area under the ROC curves (AUCs) of 0.877, 0.902 and 0.941, with the accuracy of 79.78%, 89.86% and 89.89%, respectively. In validation cohorts, the three models achieved AUCs of 0.845, 0.881 and 0.910, with the accuracy of 78.46%, 83.08% and 87.69%, respectively.

**Conclusions:**

Texture analysis based on ADC maps shows more potential than HR-T2WI in identifying preoperative T stage in rectal carcinomas. The combined application of HR-T2WI and ADC maps may help to improve the accuracy of preoperative diagnosis of rectal cancer invasion.

## Introduction

Colorectal cancer is the second most common cancer in females and the third most common cancer in males in the United States, and 30–35% of colorectal cancers occur in the rectum (1, 2). In recent years, the incidence and mortality of rectal cancer have gradually increased, showing the imbalance both in age and region, and because of the occult onset of early rectal cancer, most patients are already in the locally advanced stage at the first diagnosis (3, 4).

Surgical resection has been considered the standard treatment for patients with early rectal cancer (stages T1–2), while locally advanced stage (stages T3–4) requires total mesorectal excision (TME) after neoadjuvant chemoradiotherapy (NCRT) (5). Thus, the precise preoperative stage of rectal cancer is vitally important. However, there are challenges in diagnosing whether the stage is T2 or T3, because the perirectal desmoplastic fibrotic response is similar with tumor penetration through the muscular rectal wall, which blurs the tumor borders (6). As the gold standard for disease diagnosis, the pathological result is usually obtained after surgery and cannot be used as a routine method to guide clinical diagnosis and treatment (7). Therefore, a comprehensive noninvasive method is desired to make the preoperative risk stratification available and dependable.

As a routine examination of rectal cancer, magnetic resonance imaging (MRI) has been widely accepted as the main examination method for preoperative diagnosis of rectal cancer, selection of treatment methods, and postoperative efficacy evaluation (8–10). High-resolution T2-weighted imaging (HR-T2WI) is currently a routine sequence for MRIs to check the degree of rectal tumor invasion (11, 12). Neoplasms show a slightly higher signal than normal rectal tissue on T2WI images. Previous studies have suggested that HR-T2WI, which allows higher spatial or temporal resolution acquisitions and consequently has better signal-to-noise ratio, has better accuracy in distinguishing tumors from normal rectal tissues and diagnosing preoperative T stages of rectal cancers (13, 14).

Apparent diffusion coefficient (ADC) maps were calculated from diffusion-weighted imaging (DWI), which obtained image contrast based on differences in the mobility of water protons between tissues with two different *b* values. ADC values have been approved to be accurate in the discrimination of benign and malignant lesions and tumor assessment (15), and a lower value shows the denser cell structure of the corresponding area. A recent study showed that this quantitative index may reflect the invasion of tumor tissue into normal tissue in patients with rectal cancers (16).

Texture analysis, one of the “radiomics” aspects, is a tool for high-throughput extraction and analysis of quantitative features obtained from medical images, including computed tomography, MRI, or positron emission tomography (17, 18). This technique facilitates the prediction of tumor stage and aggressiveness, which provides a more objective method to support individual treatment options (19). As a radiology signature, the potential benefit of texture analysis has been highlighted in many studies involving clinical diagnosis, therapy selection, treatment response assessment, and so on (20–23).

However, to the best of our knowledge, the number of studies on the texture analysis of rectal ADC maps is small. Meanwhile, in most of the past studies, ROI delineations were mostly carried out on the slice images that showed the largest tumor dimension (6, 24). This study was to extract texture features from the whole tumor volume based on HR-T2WI images and ADC maps, and evaluate the performance of classification models established by three-dimensional (3D) features in predicting the preoperative T stage (stages T1-2 *versus* T3-4) in rectal carcinomas.

## Materials and Methods

**Figure 1:**
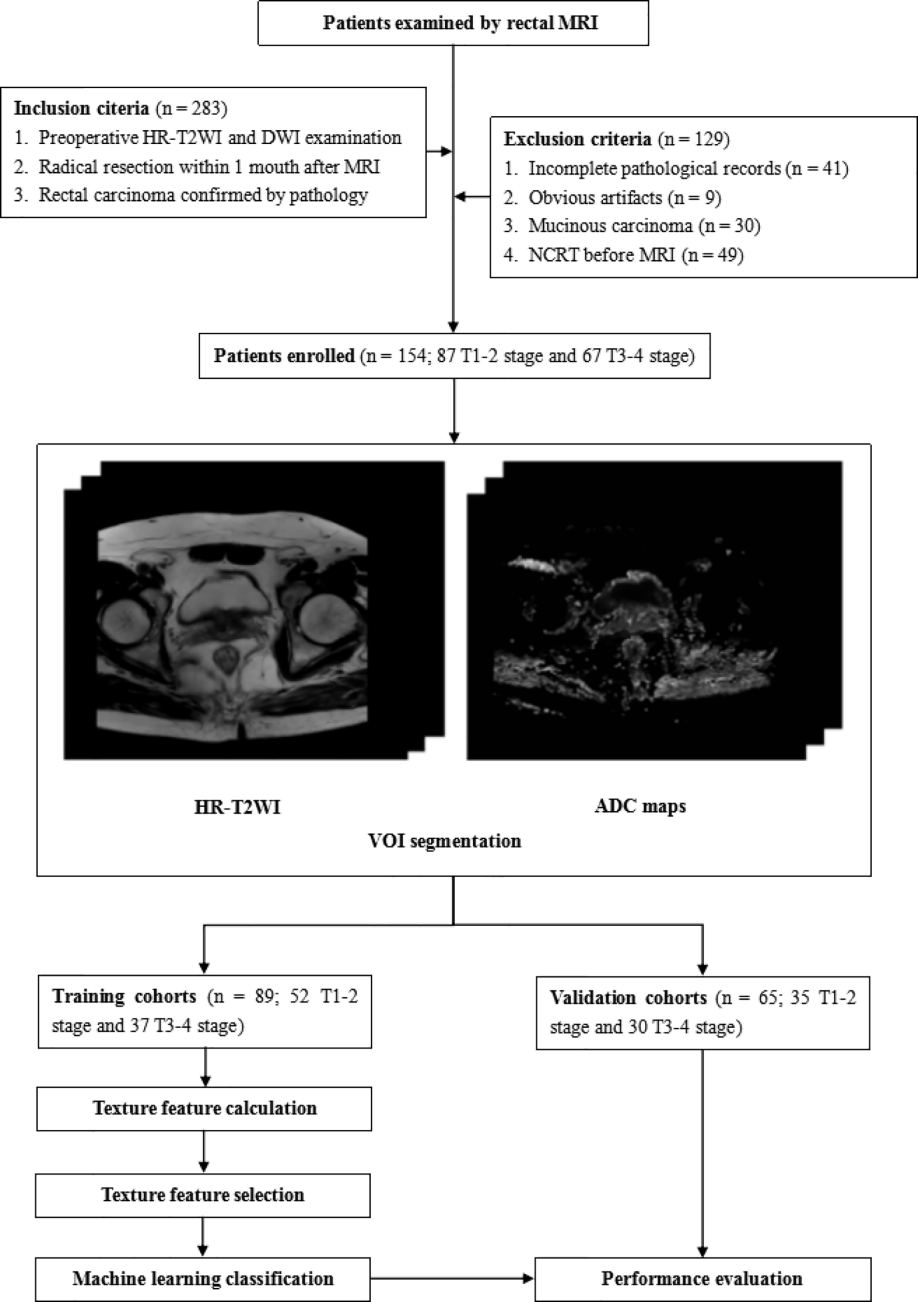
The flow chart of this research. VOI, volume of interest.

This study was approved by the Ethics Review Board of Shengjing Hospital of China Medical University (2020PS011K). Requirements for written informed consent were waived because of the retrospective nature of the study. The flow chart of this research is shown in **Figure 1**.

### Patient Cohort

Between April 2014 and December 2019, the records of 538 patients who underwent rectal MRI were reviewed using the picture archiving and communication system. A total of 283 patients were initially selected using the following inclusion criteria: 1) patients who underwent preoperative rectal HR-T2WI and DWI examinations, and 2) who underwent radical rectal cancer surgery within 1 month after MRI, 3) patients with postoperative pathology involving confirmed rectal carcinomas. One hundred and twenty-nine patients were excluded for the following reasons: 1) incomplete medical records (such as a lack of T staging information) (n = 41), 2) obvious artifacts in the images (n = 9), 3) patients with mucinous carcinoma (such patients having low cell density and high ADC values that may cause larger deviations in the results) (n = 30), and 4) patients who received NCRT before MRI examinations (n = 49). Finally, 154 patients were enrolled in this study. These patients were divided in a training cohort (n = 89; 60 males, 29 females; mean age, 61.5 ± 11.2 years; range, 26–85 years) and a validation cohort (n = 65; 44 males, 21 females; mean age 61.2 ± 11.3; range, 27–82) by different MRI scanners. In each cohort, the patients were divided into a low T stage (stages T1–2) group and a high T stage (stages T3–4) group according to pathological results.

### MRI Image Acquisition

The patients lay on the scanning bed in a supine position without bowel preparation or intravenous injection of antispasmodics. An axial HR-T2WI sequence and axial DWI sequence were conducted during the MRI examination, using an eight-channel phased array surface coil. Eighty-nine patients were examined with a 3.0 Tesla (T) MRI scanner (Ingenia 3.0; Philips Medical System, Best, The Netherlands). The acquisition parameters were as follows: repetition time/echo time, 6,000/76 ms; flip angle, 90°; matrix size, 576 × 576; field of view, 450 mm; slices, 48; slice thickness, 5 mm; spacing between slices, 1 mm; *b* values, 0 and 600 s/mm^2^. Sixty-five patients were imaged with another 3.0T MRI scanner (Signa HDxt, GE Healthcare). The acquisition parameters were as follows: repetition time/echo time, 6540/130 ms; flip angle, 90°; matrix size, 320 × 320; field of view, 360 mm; slices, 45; slice thickness, 5 mm; spacing between slices, 1 mm; b values, 0 and 600 s/mm^2^.

### ADC Map Acquisition

ADC maps were obtained with MATLAB R2018b (Mathworks, Natick, MA, USA) based on DWI images and calculated using the formula:

$$ADC=(\ln SI_{0}-\ln SI_{1})/(b-b_{0}),$$

where SI_0_ and SI_1_ represent the signal intensity of the pixel when the *b* value is 0 and 600 s/mm^2^, respectively.

### Lesion Segmentation

For each patient, two radiologists with more than 10 years of experience in rectal MRI took part in determining the tumor area using HR-T2WI with the DWI images as references. They individually and manually segmented the entire volume of interest (VOI) on HR-T2WI images, slice by slice, using 3D Slicer (version 4.10.2, www.slicer.org). Both of the two radiologists were blinded to the pathological results. Obvious lumen content areas, necrosis, and gas were excluded from subsequent analysis. The delineated VOIs based on the HR-T2WI images were exactly copied to the same location of ADC maps.

### Texture Extraction and Selection

The VOIs were first reconstructed into 3D labels and the reconstructed labels were placed on HR-T2WI and ADC maps to calculate features, and both of the two images were smoothed by the 8 mm Laplacian of Gaussian filter (25). A plug-in from 3D Slicer, Pyradiomics, was used for feature extraction. The extracted features were classified into the following categories: 1) shape-based features, 2) first-order statistics, 3) gray level dependence matrix (GLDM) features, 4) gray level co-occurrence matrix (GLCM) features, 5) gray level run length matrix features (GLRLM), 6) gray level size zone matrix (GLSZM) features, and 7) neighborhood gray-tone difference matrix (NGTDM) features. Features other than shape-based features were extracted from wavelet, Laplacian of Gaussian (Log), and original images. For wavelet transforms, each image was transformed in the x, y, and z directions using a low band pass filter or a high band pass filter. For Log transforms, the sigma (σ) values were 2.0, 3.0, 4.0, and 5.0 mm, respectively. As a result, for each patient, 1226 features were derived from HR-T2WI and ADC maps.

The above features were screened using MATLAB R2018b. First, highly correlated features were removed with coefficients greater than 0.95 using Pearson’s correlation analysis. Then, the least absolute shrinkage and selection operator (LASSO) was used to reduce the dimensionality of the remaining features with 10-fold cross-validation to avoid the overfitting. Finally, the optimal subsets of features selected by LASSO with 10 fold cross validation were statistically tested to select significant features.

### Statistical Analysis

All statistical analyses were performed using SPSS statistical software for Windows (IBM, Armonk, NY, USA), and *P* < 0.05 was considered statistically significant. The chi-square test was performed on categorical variables between different T stage groups. The Kolmogorov-Smirnov test was first used to check whether quantitative variables satisfied a normal distribution. If it satisfied a normal distribution, the independent sample *t*-test was performed between low and high T stage groups. Otherwise, the Mann-Whitney U test was performed. A support vector machine (SVM) method with 10 fold cross validation was used to establish a classification model based on the statistically significant texture features. Spearman’s correlation analysis was conducted to assess the correlation between features and tumor T stage. Multivariate logistic regression analysis was also used to evaluate whether the statistically significant features were independent predictors of T3–4 rectal tumors. The performance of classification models in predicting the different T stages of rectal tumors was evaluated using receiver operating characteristic curve analyses by Medcalc (version 14.10.20, www.medcalc.org) by measuring the area under the ROC curve (AUC). In addition, the corresponding sensitivity and specificity were also determined.

The intraclass correlation coefficients (ICCs) were calculated to evaluate the interobserver variability between two radiologists delineating the VOIs and extracting features (0–0.4, poor agreement; 0.41–0.6, moderate agreement; 0.61–0.8, good agreement; and 0.81–0.9, excellent agreement).

## Results

### Clinical Characteristics Analysis

**Figure 2:**
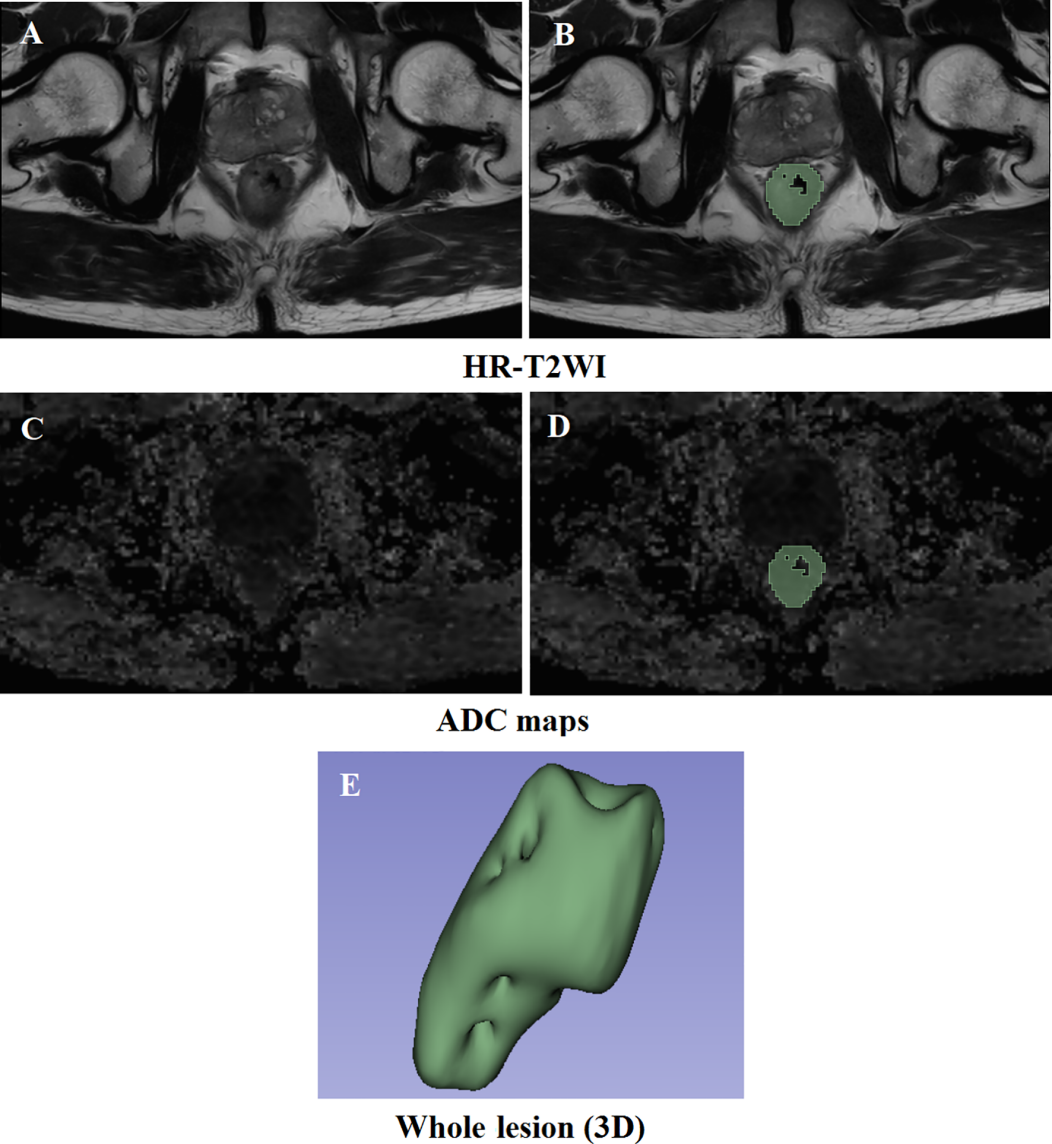
Results of the delineation of the VOI. **(A, C)** High-resolution T2-weighted imaging and apparent diffusion coefficient maps at the same slice level, respectively. **(B, D)** The result of lesion segmentation. The green marked part is the delineated lesion. **(E)** The three-dimensional label of the whole tumor.

A case was randomly selected to display the results of the VOI segmentation, as shown in **Figure 2**. The clinical and pathological characteristics of the cases selected in this study are shown in **Table 1**. There was no significant difference between low and high T stage groups in the terms of age (*P* = 0.589), sex (*P* = 0.980), and location of the lesion (*P* = 0.083). Lymph node invasion showed a significant difference between the two groups (*P* = 0.008).

**Table 1:** Clinical and pathological characteristics of the cases selected for analysis.

Characteristics	Training cohort		*Validation cohort*		*P* value
	T1-2	T3-4	T1-2	T3-4	
**Total patients**	52 (58.43%)	37 (41.57%)	35 (53.85%)	30 (46.15%)	
**Age (years)**	61.29 ± 9.645	59.97 ± 13.268	58.86 ± 13.425	61.91 ± 10.262	0.589^a^
**Sex**					0.980^b^
Male	35 (67.31%)	25 (67.57%)	24 (68.57%)	20 (66.67%)	
Female	17 (32.69%)	12 (32.43%)	11 (31.43%)	10 (33.33%)	
**Tumor location**					0.083^b^
Proximal rectum	16 (30.77%)	12 (32.43%)	10 (28.57%)	10 (33.33%)	
Middle rectum	19 (36.54%)	15 (40.54%)	13 (37.14%)	12 (40.00%)	
Distal rectum	17 (32.69%)	10 (27.03%)	12 (34.19%)	8 (26.67%)	
**Lymph node invasion**					0.008^b^
Negative	44 (84.62%)	22 (59.46%)	26 (74.29%)	16 (53.33%)	
Positive	8 (15.38%)	15 (40.54%)	9 (25.71%)	14 (46.67%)	

a Independent sample t-test was used;

b Chi-square test was used.

Date are number (%) or mean ± Standard deviation.

### Interobserver Agreement Evaluation

Features derived from the VOIs segmented separately by two radiologists showed excellent agreement, and the ICCs ranged from 0.837 to 0.945.

### Features Extracted From HR-T2WI

**Figure 3:**
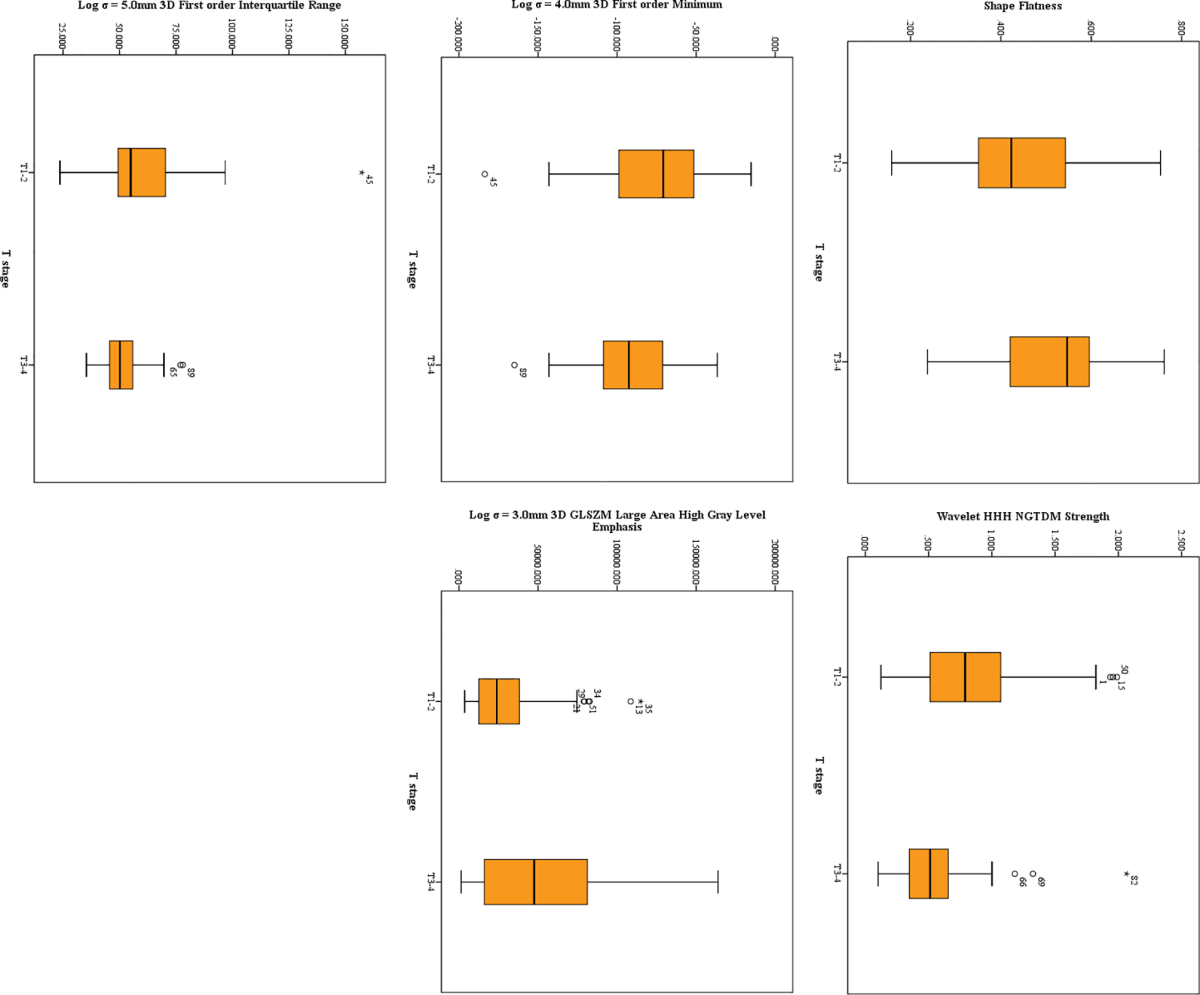
The distribution of significant features derived from HR-T2WI. The symbol (“*”) represents the extreme outlier.

Twenty features were selected from all the texture features extracted from HR-T2WI images by LASSO method. Five significant features were obtained by statistical analysis, as shown in **Table 2**. Shape flatness (*P* = 0.020), wavelet HHH NGTDM strength (*P* < 0.001), Log σ = 4.0mm 3D firstorder minimum (*P* = 0.041), Log σ = 3.0mm 3D GLSZM large area high gray level emphasis (*P* = 0.018) and Log σ = 5.0mm 3D firstorder interquartile range (*P* = 0.019) showed the significant difference in the discrimination of preoperative T stage, and achieved AUCs of 0.659, 0.713, 0.640, 0.643 and 0.646, respectively, whereas the other features were not significantly different. The distribution of those features is shown in **Figure 3**. The results of multivariate logistic regression analysis showed that the wavelet HHH NGTDM strength was an independent predictor of stage T3-4 rectal tumors, and it showed the highest correlation (*R_S_* = -0.364, *P* < 0.001) with the preoperative T stage of rectal cancer among the significant features.

**Table 2:** Significant features between stage T1–2 and T3–4 tumors derived from HR-T2WI.

Feature	T stage		AUC	*R_S_*	*P* value
	T1-2 (n = 52)	T3-4 (n = 37)			
Shape Flatness	0.444 ± 0.139	0.513 ± 0.131	0.659	0.247	0.020^a^
Wavelet HHH	0.788 ± 0.582	0.512 ± 0.309	0.713	-0.364	<0.001^b^
NGTDM Strength			
Log σ = 4.0mm 3D	-76.35 ± 33.73	-90.52 ± 28.83	0.640	0.249	0.041^a^
Firstorder Minimum			
Log σ = 3.0mm 3D GLSZM Large Area High Gray Level Emphasis	23925.256 ± 26104.701	47535.971 ± 65621.849	0.643	-0.249	0.018^b^
Log σ = 5.0mm 3D	54.943 ± 21.370	50.147 ± 12.872	0.646	-0.217	0.019^b^
Firstorder Interquartile Range			

a Independent sample t-test was used, and data are the mean ± SD;

b Mann–Whitney U test was used, data are the medians ± interquartile range.

AUC, area under the receiver operating characteristic curve; NGTDM, neighborhood gray-tone difference matrix; Log, Laplacian of Gaussian; GLSZM, gray level size zone matrix.

### Features Extracted From ADC Maps

**Figure 4:**
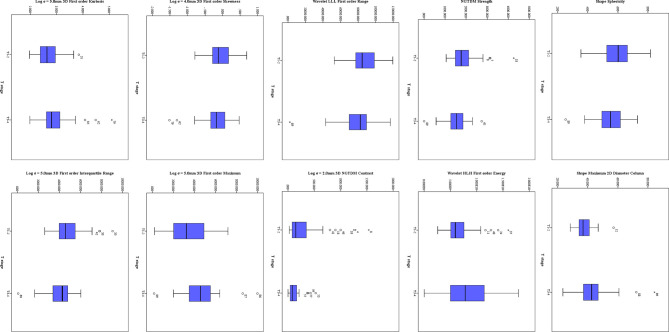
The distribution of significant features derived from ADC maps. The symbol (“*”) represents the extreme outlier.

Twenty-three features were selected from all the texture features extracted from ADC maps by LASSO method. Ten significant features were obtained by statistical analysis, as shown in **Table 3**. Shape sphericity (*P* = 0.027), shape maximum 2DDiameter Column (*P* < 0.001), NGTDM Strength (*P* = 0.018), wavelet HLH first order energy (*P* = 0.049), wavelet LLL first order range (*P* = 0.024), Log σ = 2.0 mm 3D NGTDM contrast (*P* = 0.003), Log σ = 4.0 mm 3D firstorder skewness (*P* = 0.024), Log σ = 5.0 mm 3D first order maximum (*P* = 0.009), Log σ = 5.0 mm 3D first order kurtosis (*P* = 0.011) and Log σ = 5.0 mm 3D first order interquartile range (*P* = 0.032) showed the significant difference in the discrimination of preoperative T stage, and achieved AUCs of 0.641, 0.752, 0.647, 0.623, 0.614, 0.683, 0.598, 0.659, 0.659 and 0.634, respectively, whereas other features did not present significant difference. The results of multivariate logistic regression analysis showed that the shape maximum two-dimensional (2D) diameter column, Log σ = 5.0 mm 3D first-order maximum and Log σ = 5.0 mm 3D first-order interquartile range were independent predictors of stage T3–4 tumors. The shape maximum 2D diameter column showed the highest correlation with the preoperative T stage (*R_S_* = 0.431, *P* < 0.001). The distribution of significant features from ADC maps is shown in **Figure 4**.

**Table 3:** Significant features between stage T1–2 and T3–4 tumors derived from ADC maps.

Feature	T stage		AUC	*R_S_*	*P* value
	T1-2 (n = 52)	T3-4 (n = 37)			
Shape Sphericity	0.600 ± 0.112	0.547 ± 0.106	0.641	-0.235	0.027^a^
Shape Maximum	36.864 ± 7.143	42.638 ± 11.033	0.752	0.431	<0.001^b^
2D Diameter Column			
NGTDM Strength	1768.469 ± 631.326	1546.459 ± 600.620	0.647	-0.251	0.018^b^
Wavelet HLH	6534962116 ± 2544354482	7850582575 ± 6464968925	0.623	0.210	0.049^b^
First order Energy			
Wavelet LLL	86766.186 ± 14721.532	78306.717 ± 20030.935	0.614	-0.239	0.024^a^
First order Range			
Log σ = 2.0 mm 3D	148.315 ± 272.076	81.591 ± 127.352	0.683	-0.313	0.003^b^
NGTDM Contrast			
Log σ = 4.0 mm 3D	-0.048 ± 0.318	-0.225 ± 0.409	0.598	-0.239	0.024^a^
Firstorder Skewness			
Log σ = 5.0 mm 3D	3466.430 ± 1879.743	4534.088 ± 1810.633	0.659	0.276	0.009^a^
First order Maximum			
Log σ = 5.0 mm 3D	2.637 ± 0.579	2.811 ± 0.617	0.659	0.272	0.011^b^
First order Kurtosis			
Log σ = 5.0 mm 3D	4557.574 ± 1560.537	4270.367 ± 1436.946	0.634	-0.229	0.032^b^
First order Interquartile Range			

a Independent sample t-test was used, and data are the mean ± SD;

b Mann–Whitney U test was used, data are the medians ± interquartile range.

AUC, area under the receiver operating characteristic curve; NGTDM, neighborhood gray-tone difference matrix; Log, Laplacian of Gaussian.

### Performance of Classification Models

**Figure 5:**
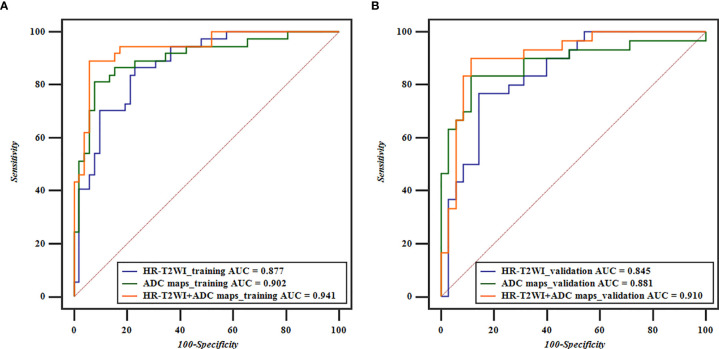
ROC curves based on HR-T2WI and ADC maps for predicting preoperative T1-2 and T3-4 stage of rectal cancer. **(A)** The ROC curves of the training cohorts of T stage, and **(B)** the ROC curves of the validation cohorts of T stage.

The performance of classification models for identifying preoperative rectal cancer T stage was shown in **Table 4**. In training cohorts, the model from HR-T2WI achieved an AUC of 0.877 [95% confidence interval (CI), 0.791-0.937] with a sensitivity of 86.54%, a specificity of 78.38%, and an accuracy of 79.78%. The model from ADC maps achieved an AUC of 0.902 (95% CI, 0.820-0.955) with a sensitivity of 81.08%, a specificity of 92.31%, and an accuracy of 89.86%. The combination model from HR-T2WI and ADC maps achieved an AUC of 0.941 (95% CI, 0.870-0.980) with a sensitivity of 89.19%, a specificity of 94.23%, and an accuracy of 89.89%. In validation cohorts, the model from HR-T2WI achieved an AUC of 0.845 (95% CI, 0.734-0.923) with a sensitivity of 76.67%, a specificity of 85.71%, and an accuracy of 78.46%. The model from ADC maps achieved an AUC of 0.881 (95% CI, 0.777-0.948) with a sensitivity of 83.33%, a specificity of 88.57%, and an accuracy of 83.08%. The combination model from HR-T2WI and ADC maps achieved an AUC of 0.910 (95% CI, 0.812-0.966) with a sensitivity of 90.00%, a specificity of 88.57%, and an accuracy of 87.69%. The ROC curves of LASSO_SVM models established by features extracted from HR-T2WI and ADC maps are shown in **Figure 5**.

**Table 4:** Performance of classification models for identifying preoperative T stage of rectal cancer.

Methods		Cohorts	AUC	95% CI	Sensitivity (%)	Specificity (%)	Accuracy (%)
**T stage**	**HR-T2WI**	Training	0.877	0.791-0.937	86.54	78.38	79.78
		Validation	0.845	0.734-0.923	76.67	85.71	78.46
	**ADC maps**	Training	0.902	0.820-0.955	81.08	92.31	89.86
		Validation	0.881	0.777-0.948	83.33	88.57	83.08
	**Combination**	Training	0.941	0.870-0.980	89.19	94.23	89.89
		Validation	0.910	0.812-0.966	90.00	88.57	87.69

Combination means the joint application of classification models based on HR-T2WI and ADC maps.

## Discussion

In the present study, 3D VOI texture analysis was applied to rectal HR-T2WI and ADC maps, and the correlations between the whole tumor volume features and T stage were investigated. The results showed that texture features played a potential role in predicting the preoperative T stage of rectal carcinomas.

Preoperative staging are essential for treatment choices and prognosis evaluations of patients with rectal cancers. Especially for locally advanced patients, NCRT is required before accepting TME to ensure smooth progress of the operation and a better treatment response (5). In fact, the differential diagnosis of stages T2 and T3 is still difficult due to the perirectal desmoplastic fibrotic response (6). Texture analysis can describe the relationship between the gray level intensity of pixels and quantify the heterogeneity in images (18, 26, 27). And MRI-derived texture parameters have been proposed as tools for accurate diagnosis, preoperative risk stratification, or assessment of treatment response in several cancer types, including tumors in the brain, breast, prostate, and uterus (28–31). As reported in previous studies, high-resolution MRI has higher soft tissue resolution and can help in the diagnosis and stage-oriented treatment decisions (32–36). He et al. (25) suggested that MRI-based radiomic signatures showed acceptable performance for tumor grading of rectal carcinomas. Sun et al. (34) verified the feasibility of using radiomic features from T2WI images to identify the T staging of rectal cancer. Meanwhile, texture analysis of ADC maps was proven to have potential in cancer diagnosis, such as breast carcinoma (37), Cervical Carcinoma (38), Renal Carcinoma (39), gliomas (40). A study from Liu et al. (6) indicated that texture analysis on the features from the single slice of ADC map that showed the largest tumor dimension could provide valuable information in identifying locally advanced rectal cancer. However, few studies have focused on the 3D features extracted from whole volume lesion from HR-T2WI and ADC maps to distinguish the preoperative T stage of rectal cancers. Our study conducted the volume texture analysis based on HR-T2WI or ADC maps.

In this study, a total of 1226 texture features were calculated from preoperative HR-T2WI and ADC maps. These features were selected by LASSO to obtain optimum feature subsets, and statistical analysis was carried out to further reduce feature dimensionality. The classification models were established using the SVM method, which has been proven to have better performance and widely used in previous texture analysis reports (41). In training cohorts, the accuracy of the LASSO_SVM models based on HR-T2WI and ADC maps was 79.78% and 89.86% in predicting T stage of rectal cancers. The corresponding AUC values were 0.877 and 0.902, respectively. In validation cohorts, the accuracy of the LASSO_SVM models based on HR-T2WI and ADC maps was 78.46% and 83.08% in predicting T stage of rectal cancers. The corresponding AUC values were 0.845 and 0.881, respectively. The results demonstrated that pretreatment HR-T2WI and ADC maps had high potential application in identifying the preoperative T stage of rectal cancers, which is consistent with the results of Sun et al. (6, 34, 42). Meanwhile, the performance of the 3D features from ADC maps showed better than that of features from HR-T2WI. In the training cohort, the accuracy of the joint model of HR-T2WI and ADC maps was 89.89% with AUC of 0.941. In the validation cohort, the accuracy of the joint model was 87.69% with AUC of 0.910. It could be concluded that the combination model of HR-T2WI and ADC maps presented better performance than that using each of them alone. Cui et al. (43) established a radiomics predictive model based on pre-treatment multiparameter features from pre-CRT T2-weighted (T2-w), contrast-enhanced T1-weighted (cT1-w) and ADC maps and clinical features to predict a pathological complete response (pCR) in patients with locally advanced rectal cancer. They pointed that 3D features from ADC maps showed better performance than T2-w and cT1-w, and the radiomics signatures from joint T2-w, ADC and cT1-w images achieved better predictive efficacy than those from any of them alone. Their opinion was similar to ours.

There are controversies regarding the use of ADC values in predicting preoperative rectal T stages (6, 16, 44). In the present study, ADC quantification calculated from original images was not statistically significant between different T stages, which is consistent with the results of Liu et al. (6). However, the maximum of ADC value was statistically significant when a Log transform of σ = 5.0 mm was used. It may be because the conventional measurement of the ADC is only a mean value of signal intensity, and the heterogeneous intensity may be weakened due to the mutual influence of signals between various tissues. Compared with routine ADC measurements, texture analysis on ADC maps may therefore better predict local invasion of rectal cancers.

He et al. (25) indicated that not all categories of radiomics features contributed equally in the grading of rectal cancer. The transformed first-order features showed more significance than higher order features, and could diagnose and predict the grading of rectal carcinoma relatively, steadily and accurately (25). This point was similar to that of our study. After the original image was transformed by wavelet and Log, the parameters related to the lesion in the original image may be emphasized. Therefore, the transformed features may have better abilities to identify lesions than the original features, and were retained after screening. Cui et al. (45) develop a T2-weighted image-based radiomics signature for the individual prediction of KRAS mutation status in patients with rectal cancer. Most of the obtained features were from the images filtered by wavelet or log, and many of them appeared to be shape and first order with fewer higher order features. That point was similar to ours.

In addition, interobserver variability in feature calculations between two radiologists was evaluated. The values of ICCs ranging from 0.837 to 0.945, showed excellent agreement. The interobserver variability mainly occurred in delineating the lesion region slice by slice. Thus, it is important to reach a consensus between two radiologists in determining the tumor areas on HR-T2WI and ADC maps.

This study had some limitations. First, texture analysis was usually based on a large number of samples. Therefore, there may be potential biases because of the small sample in this study. Second, stage T2 and T3 accounted for a larger proportion among the enrolled cases, while stage T1 and T4 accounted for a smaller proportion. This study only focused on the differential diagnosis between early stage and local advanced stage, and divided patients into low (stages T1–2) and high (stages T3–4) T stage groups. More cases need to be collected, especially stage T1 and T4 tumors. Finally, whole tumor VOI delineation is a time-consuming operation. Two radiologists who were familiar with the operation of 3D Slicer spent an average of 15 minutes to outline and identify the whole lesion, which played a negative role in the progress and application of texture analysis.

In conclusion, texture features with LASSO_SVM models had good performance in predicting local invasion of rectal cancer. Texture analysis based on ADC maps was more potential than that based on HR-T2WI in identifying preoperative T stage in rectal carcinomas. The combined application of HR-T2WI and ADC maps could be used as an auxiliary diagnostic option for preoperative diagnosis of rectal cancer invasion.

## Data Availability Statement

The raw data supporting the conclusions of this article will be made available by the authors, without undue reservation.

## Ethics Statement

This study was approved by the Ethics Review Board of Shengjing Hospital of China Medical University (2020PS011K). Requirements for written informed consent were waived because of the retrospective nature of the study.

## Author Contributions

JY analyzed the patient data, and wrote the paper. JDY was a major contributor in designing the manuscript. All authors contributed to the article and approved the submitted version.

## Funding

This research was supported by grants from the Research and Development (R&D) Foundation for Major Science and Technology from Shenyang (No. 19-112-4-105), the Big Data Foundation for Health Care from China Medical University (No. HMB201902105), the Natural Fund Guidance Plan from Liaoning (No. 2019-ZD-0743), and 345 talent project from Shengjing Hospital.

## Conflict of Interest

The authors declare that the research was conducted in the absence of any commercial or financial relationships that could be construed as a potential conflict of interest.

## Publisher’s Note

All claims expressed in this article are solely those of the authors and do not necessarily represent those of their affiliated organizations, or those of the publisher, the editors and the reviewers. Any product that may be evaluated in this article, or claim that may be made by its manufacturer, is not guaranteed or endorsed by the publisher.

## References

[1] Siegel RL Miller KD Jemal A. Cancer Statistics, 2020. CA Cancer J Clin (2020) 70(1):7–30. 10.3322/caac.21590 31912902

[2] Glynne-Jones R Wyrwicz L Tiret E Brown G Rödel C Cervantes A. Rectal Cancer: ESMO Clinical Practice Guidelines for Diagnosis, Treatment and Follow-Up. Ann Oncol (2018) 29(Suppl 4):iv263. 10.1093/annonc/mdy161 29741565

[3] Saule B Sajad AB Arip K Zhanna G Olzhas U Anar T. Trends in Colorectal Cancer Incidence in Western Kazakhstan Through the First Decade of the Screening Implementation, 2009–2018. J Coloproctol (2020) 40(1):43–9. 10.1016/j.jcol.2019.10.004

[4] Bray F Ferlay J Soerjomataram I Siegel RL Torre LA Jemal A. Global Cancer Statistics 2018: GLOBOCAN Estimates of Incidence and Mortality Worldwide for 36 Cancers in 185 Countries. CA Cancer J Clin (2018) 68(6):394–424. 10.3322/caac.21492 30207593

[5] Goldenberg BA Holliday EB Helewa RM Singh H. Rectal Cancer in 2018: A Primer for the Gastroenterologist. Am J Gastroenterol (2018) 113(12):1763–71. 10.1038/s41395-018-0180-y PMC6768608 30008472

[6] Liu L Liu Y Xu L Li Z Lv H Dong N. Application of Texture Analysis Based on Apparent Diffusion Coefficient Maps in Discriminating Different Stages of Rectal Cancer. J Magn Reson Imaging (2017) 45(6):1798–808. 10.1002/jmri.25460 27654307

[7] Rania AES Mohammad A Mohammad EK. Preoperative MRI Evaluation of Mesorectum in Cases of Rectal Carcinoma. J Menoufia Med J (2017) 30(1):122–7. 10.4103/1110-2098.211484

[8] Benson AB Venook AP Al-Hawary MM Cederquist L Chen YJ Ciombor KK. Nccn Guidelines Insights: Colon Cancer, Version 2.2018. J Natl Compr Canc Netw (2018) 16(4):359–69. 10.6004/jnccn.2018.0021 PMC10184502 29632055

[9] Zhang XM Zhang HL Yu D Dai Y Bi D Prince MR. 3-T MRI of Rectal Carcinoma: Preoperative Diagnosis, Staging, and Planning of Sphincter-Sparing Surgery. AJR Am J Roentgenol (2008) 190(5):1271–8. 10.2214/AJR.07.2505 18430843

[10] Shaish H Aukerman A Vanguri R Spinelli A Armenta P Jambawalikar S. Radiomics of MRI for Pretreatment Prediction of Pathologic Complete Response, Tumor Regression Grade, and Neoadjuvant Rectal Score in Patients With Locally Advanced Rectal Cancer Undergoing Neoadjuvant Chemoradiation: An International Multicenter Study. Eur Radiol (2020) 30(11):6263–73. 10.1007/s00330-020-06968-6 32500192

[11] Feng Q Yan YQ Zhu J Xu JR. T Staging of Rectal Cancer: Accuracy of Diffusion-Weighted Imaging Compared With T2-Weighted Imaging on 3. 0 tesla MRI. J Dig Dis (2014) 15(4):188–94. 10.1111/1751-2980.12124 24373561

[12] Chen XL Chen GW Pu H Yin LL Li ZL Song B. DWI and T2-Weighted MRI Volumetry in Resectable Rectal Cancer: Correlation With Lymphovascular Invasion and Lymph Node Metastases. AJR Am J Roentgenol (2019) 212(6):1271–8. 10.2214/AJR.18.20564 30933653

[13] Hoogendam JP Kalleveen IM de Castro CS Raaijmakers AJ Verheijen RH van den Bosch MA. High-Resolution T2-Weighted Cervical Cancer Imaging: A Feasibility Study on Ultra-High-Field 7.0-T MRI With an Endorectal Monopole Antenna. Eur Radiol (2017) 27(3):938–45. 10.1007/s00330-016-4419-y PMC5306309 27246722

[14] Kuhl CK Träber F Schild HH. Whole-Body High-Field-Strength (3.0-T) MR Imaging in Clinical Practice. Part I. Technical Considerations and Clinical Applications. Radiology (2008) 246(3):675–96. 10.1148/radiol.2463060881 18309012

[15] Iannicelli E Di Pietropaolo M Pilozzi E Osti MF Valentino M Masoni L. Value of Diffusion-Weighted MRI and Apparent Diffusion Coefficient Measurements for Predicting the Response of Locally Advanced Rectal Cancer to Neoadjuvant Chemoradiotherapy. Abdom Radiol (NY) (2016) 41(10):1906–17. 10.1007/s00261-016-0805-9 27323759

[16] Curvo-Semedo L Lambregts DM Maas M Beets GL Caseiro-Alves F Beets-Tan RG. Diffusion-Weighted MRI in Rectal Cancer: Apparent Diffusion Coefficient as a Potential Noninvasive Marker of Tumor Aggressiveness. J Magn Reson Imaging (2012) 35(6):1365–71. 10.1002/jmri.23589 22271382

[17] Lambin P Rios-Velazquez E Leijenaar R Carvalho S van Stiphout RG Granton P. Radiomics: Extracting More Information From Medical Images Using Advanced Feature Analysis. Eur J Cancer (2012) 48(4):441–6. 10.1016/j.ejca.2011.11.036 PMC4533986 22257792

[18] Gillies RJ Kinahan PE Hricak H. Radiomics: Images are More Than Pictures, They are Data. Radiology (2016) 278(2):563–77. 10.1148/radiol.2015151169 PMC4734157 26579733

[19] Liu H Zhang C Wang L Luo R Li J Zheng H. MRI Radiomics Analysis for Predicting Preoperative Synchronous Distant Metastasis in Patients With Rectal Cancer. Eur Radiol (2019) 29(8):4418–26. 10.1007/s00330-018-5802-7 30413955

[20] Fu J Fang MJ Dong D Li J Sun YS Tian J. Heterogeneity of Metastatic Gastrointestinal Stromal Tumor on Texture Analysis: DWI Texture as Potential Biomarker of Overall Survival. Eur J Radiol (2020) 125:108825. 10.1016/j.ejrad.2020.108825 32035324

[21] Corino VDA Montin E Messina A Casali PG Gronchi A Marchianò A. Radiomic Analysis of Soft Tissues Sarcomas can Distinguish Intermediate From High-Grade Lesions. J Magn Reson Imaging (2018) 47(3):829–40. 10.1002/jmri.25791 28653477

[22] Meng Y Zhang Y Dong D Li C Liang X Zhang C. Novel Radiomic Signature as a Prognostic Biomarker for Locally Advanced Rectal Cancer. J Magn Reson Imaging (2018) 48(3):605–14. 10.1002/jmri.25968 29437271

[23] Zhang Y Oikonomou A Wong A Haider MA Khalvati F. Radiomics-Based Prognosis Analysis for non-Small Cell Lung Cancer. Sci Rep (2017) 7:46349. 10.1038/srep46349 28418006 PMC5394465

[24] Yin JD Song LR Lu HC Zheng X. Prediction of Different Stages of Rectal Cancer: Texture Analysis Based on Diffusion-Weighted Images and Apparent Diffusion Coefficient Maps. World J Gastroenterol (2020) 26(17):2082–96. 10.3748/wjg.v26.i17.2082 PMC7267694 32536776

[25] He B Ji T Zhang H Zhu Y Shu R Zhao W. MRI-Based Radiomics Signature for Tumor Grading of Rectal Carcinoma Using Random Forest Model. J Cell Physiol (2019) 234(11):20501–9. 10.1002/jcp.28650 31074022

[26] Kudou M Nakanishi M Kuriu Y Murayama Y Arita T Kishimoto M. Value of Intra-Tumor Heterogeneity Evaluated by Diffusion-Weighted MRI for Predicting Pathological Stages and Therapeutic Responses to Chemoradiotherapy in Lower Rectal Cancer. J Cancer (2020) 11(1):168–76. 10.7150/jca.38354 PMC6930416 31892983

[27] Li X Zhu H Qian X Chen N Lin X. MRI Texture Analysis for Differentiating Nonfunctional Pancreatic Neuroendocrine Neoplasms From Solid Pseudopapillary Neoplasms of the Pancreas. Acad Radiol (2020) 27(6):815–23. 10.1016/j.acra.2019.07.012 31444110

[28] Han Y Wang T Wu P Zhang H Chen H Yang C. Meningiomas: Preoperative Predictive Histopathological Grading Based on Radiomics of MRI. Magn Reson Imaging (2021) 77:36–43. 10.1016/j.mri.2020.11.009 33220449

[29] Ahmed A Gibbs P Pickles M Turnbull L. Texture Analysis in Assessment and Prediction of Chemotherapy Response in Breast Cancer. J Magn Reson Imaging (2013) 38(1):89–101. 10.1002/jmri.23971 23238914

[30] Wibmer A Hricak H Gondo T Matsumoto K Veeraraghavan H Fehr D. Haralick Texture Analysis of Prostate MRI: Utility for Differentiating non-Cancerous Prostate From Prostate Cancer and Differentiating Prostate Cancers With Different Gleason Scores. Eur Radiol (2015) 25(10):2840–50. 10.1007/s00330-015-3701-8 PMC5026307 25991476

[31] Ytre-Hauge S Dybvik JA Lundervold A Salvesen ØO Krakstad C Fasmer KE. Preoperative Tumor Texture Analysis on MRI Predicts High-Risk Disease and Reduced Survival in Endometrial Cancer. J Magn Reson Imaging (2018) 48(6):1637–47. 10.1002/jmri.26184 30102441

[32] Tudyka V Blomqvist L Beets-Tan RG Boelens PG Valentini V van de Velde CJ. EURECCA Consensus Conference Highlights About Colon & Rectal Cancer Multidisciplinary Management: The Radiology Experts Review. Eur J Surg Oncol (2014) 40(4):469–75. 10.1016/j.ejso.2013.10.029 24439446

[33] KSAR Study Group for Rectal Cancer. Essential Items for Structured Reporting of Rectal Cancer MRI: 2016 Consensus Recommendation From the Korean Society of Abdominal Radiology. Korean J Radiol (2017) 18(1):132–51. 10.3348/kjr.2017.18.1.132 PMC5240498 28096724

[34] Sun Y Hu P Wang J Shen L Xia F Qing G. Radiomic Features of Pretreatment MRI Could Identify T Stage in Patients With Rectal Cancer: Preliminary Findings. J Magn Reson Imaging (2018) 48(3):615–21. 10.1002/jmri.25969 29437279

[35] Just N. Improving Tumour Heterogeneity MRI Assessment With Histograms. Br J Cancer (2014) 111(12):2205–13. 10.1038/bjc.2014.512 PMC4264439 25268373

[36] Choudhery S Gomez-Cardona D Favazza CP Hoskin TL Haddad TC Goetz MP. MRI Radiomics for Assessment of Molecular Subtype, Pathological Complete Response, and Residual Cancer Burden in Breast Cancer Patients Treated With Neoadjuvant Chemotherapy. Acad Radiol (2020) S1076-6332(20):30607–3. 10.1016/j.acra.2020.10.020 PMC8093323 33160859

[37] Zhao Q Xie T Fu C Chen L Bai Q Grimm R. Differentiation Between Idiopathic Granulomatous Mastitis and Invasive Breast Carcinoma, Both Presenting With non-Mass Enhancement Without Rim-Enhanced Masses: The Value of Whole-Lesion Histogram and Texture Analysis Using Apparent Diffusion Coefficient. Eur J Radiol (2020) 123:108782. 10.1016/j.ejrad.2019.108782 31864142

[38] Yamada I Oshima N Miyasaka N Wakana K Wakabayashi A Sakamoto J. Texture Analysis of Apparent Diffusion Coefficient Maps in Cervical Carcinoma: Correlation With Histopathologic Findings and Prognosis. Radiol Imaging Cancer (2020) 2(3):e190085. 10.1148/rycan.2020190085 33778713 PMC7983793

[39] Li H Li A Zhu H Hu Y Li J Xia L. Whole-Tumor Quantitative Apparent Diffusion Coefficient Histogram and Texture Analysis to Differentiation of Minimal Fat Angiomyolipoma From Clear Cell Renal Cell Carcinoma. Acad Radiol (2019) 26(5):632–9. 10.1016/j.acra.2018.06.015 30087067

[40] Kanazawa T Minami Y Takahashi H Fujiwara H Toda M Jinzaki M. Magnetic Resonance Imaging Texture Analyses in Lower-Grade Gliomas With a Commercially Available Software: Correlation of Apparent Diffusion Coefficient and T2 Skewness With 1p/19q Codeletion. Neurosurg Rev (2020) 43(4):1211–9. 10.1007/s10143-019-01157-6 31402410

[41] Qian Z Li Y Wang Y Li L Li R Wang K. Differentiation of Glioblastoma From Solitary Brain Metastases Using Radiomic Machine-Learning Classifiers. Cancer Lett (2019) 451:128–35. 10.1016/j.canlet.2019.02.054 30878526

[42] Ma X Shen F Jia Y Xia Y Li Q Lu J. MRI-Based Radiomics of Rectal Cancer: Preoperative Assessment of the Pathological Features. BMC Med Imaging (2019) 19(1):86. 10.1186/s12880-019-0392-7 31747902 PMC6864926

[43] Cui Y Yang X Shi Z Yang Z Du X Zhao Z. Radiomics Analysis of Multiparametric MRI for Prediction of Pathological Complete Response to Neoadjuvant Chemoradiotherapy in Locally Advanced Rectal Cancer. Eur Radiol (2019) 29(3):1211–20. 10.1007/s00330-018-5683-9 30128616

[44] Sun Y Tong T Cai S Bi R Xin C Gu Y. Apparent Diffusion Coefficient (ADC) Value: A Potential Imaging Biomarker That Reflects the Biological Features of Rectal Cancer. PLoS One (2014) 9(10):e109371. 10.1371/journal.pone.0109371 25303288 PMC4193754

[45] Cui Y Liu H Ren J Du X Xin L Li D. Development and Validation of a MRI-Based Radiomics Signature for Prediction of KRAS Mutation in Rectal Cancer. Eur Radiol (2020) 30(4):1948–58. 10.1007/s00330-019-06572-3 31942672

